# Role of CaCO_3_ in Retarding UV- and Thermally Induced Degradation of PVC Compounds

**DOI:** 10.3390/ma19153270

**Published:** 2026-08-02

**Authors:** Soraya Nait Larbi, Abdallah Hedir, Mustapha Moudoud, David Clark, Ali Durmus, Omar Lamrous, Abderrahmane Haddad

**Affiliations:** 1 Laboratoire de Technologies Avancées en Génie Electrique, (LATAGE), Université Mouloud Mammeri de Tizi-Ouzou, BP 17 RP, Tizi-Ouzou 15000, Algeria; 2 Advanced High Voltage Engineering Center, School of Engineering, Cardiff University, Queen’s Buildings, The Parade, Cardiff CF24 3AA, UK; 3 Department of Chemical Engineering, Engineering Faculty, Istanbul University-Cerrahpasa, Istanbul 34320, Turkey; 4 Laboratoire de Physique et Chimie Quantique (LPCQ), Université Mouloud Mammeri de Tizi-Ouzou, BP 17 RP, Tizi-Ouzou 15000, Algeria

**Keywords:** PVC/CaCO_3_ composites, accelerated aging, mechanical properties, surface morphology, service life

## Abstract

This study provides an in-depth investigation of the influence of calcium carbonate (CaCO_3_) filler on the mechanical performance and aging resistance of polyvinyl chloride (PVC)-based composites. PVC/CaCO_3_ composites containing 2.5, 5, and 7.5 wt% of CaCO_3_ were subjected to accelerated aging under combined ultraviolet (UV) irradiation and thermal stress for up to 1248 h. The key mechanical properties of specimens —tensile strength and elongation at break—were measured before and after aging. Changes in surface morphology, coloration, hydrophobicity, and chemical composition were characterized using scanning electron microscopy (SEM-EDS), X-ray spectroscopy, atomic force microscopy (AFM), contact angle measurements, and carbonyl index calculation to quantify relationships between the structural and physical properties of the specimens and aging conditions. The results reveal a significant correlation between filler content and the mechanical behavior of aged specimens, highlighting the potential of CaCO_3_ reinforcement not only to improve the retention of mechanical properties but also to increase the service life of PVC-based insulation compounds.

## 1. Introduction

Polymeric materials play a critical role in meeting the increasing insulation-performance requirements of high-voltage direct-current (HVDC) and alternating-current (HVAC) systems, including cables, transformers, and composite insulators, thereby significantly influencing operating voltage levels and power-transmission efficiency. The rapid expansion of renewable-energy generation, offshore transmission networks, and high-voltage and pulsed-power technologies is driving demand for polymeric insulation materials that exhibit enhanced performance, reliability, and durability under harsh operating conditions [1]. This trend reflects the ongoing transformation of the global energy sector, in which power systems are evolving toward higher voltage levels, greater operational flexibility, and deployment in increasingly demanding environments. Consequently, the reliability of insulation systems has become a critical requirement for modern high-voltage power networks, with polymeric materials offering several advantages over traditional inorganic insulating materials [2,3].

Under increasing power densities and greater system integration, insulation materials face several challenges, including premature electrical breakdown, limited thermal resistance, and space-charge accumulation, all of which can adversely affect their dielectric, electrical, structural, and mechanical properties. The future of high-voltage electrical equipment depends on the continued development and reliability of polymeric insulating materials, which offer high-voltage endurance and ease of processing, making them particularly suitable for modern electronics, high-frequency communication systems, high-power devices, and high-voltage power grids and converters [4,5].

Among the polymeric materials used in cable-insulation applications, plasticized poly(vinyl chloride) (PVC) compounds remain widely employed because of their excellent chemical resistance, flame-retardant characteristics, and cost-effectiveness. Additional advantages include (i) ease of processing, (ii) relatively low material and manufacturing costs compared with polyethylene (PE), cross-linked polyethylene (XLPE), and rubber-based insulation systems, and (iii) formulation flexibility that enables the development of application-specific compounds. It should be noted that sPVC compounds are multi-component materials comprising PVC as the base polymer, liquid plasticizers (typically high molecular weight aromatic organic esters), various stabilizers, fillers (e.g., natural and synthetic inorganic particles), and processing aids like lubricants. Depending on the application, additional additives such as migration inhibitors, pigments, and smoke suppressants may also be incorporated. Exposure to ultraviolet (UV) radiation, particularly when combined with elevated temperatures, accelerates PVC degradation through photo-oxidation, thermo-oxidation, and photolytic reactions. These degradation processes promote polymer-chain scission, microcrack formation, and discoloration, ultimately leading to deterioration of the material’s physical and mechanical properties [6,7]. Furthermore, discoloration may arise from dehydrochlorination reactions occurring during thermal exposure. To mitigate these effects, extensive research has focused on improving PVC performance through the incorporation of micro- and fillers or through polymer blending strategies. Notably, even small amounts of fillers can significantly improve the physical properties, long-term durability, and environmental stability of PVC under severe operating conditions [8,9,10,11].

To overcome these limitations, considerable research has focused on polymer nanocomposites in which micro- and nano-scale inorganic fillers are incorporated into polymer matrices. This approach has emerged as an effective strategy for inhibiting PVC photodegradation while enhancing its mechanical, thermal, and dielectric properties, thereby improving long-term service life and structural stability [12,13]. Among the fillers used in PVC formulations, calcium carbonate (CaCO_3_) is one of the most widely employed because of its abundance, low cost, non-toxicity, and favorable compatibility with polymer-processing technologies. It has long been employed as a filler in PVC formulations, as well as in other thermoplastics (e.g., polyethylene (PE), polypropylene (PP), and thermoplastic elastomers (TPEs)) owing to its proven ability to enhance the mechanical properties of polymer composites. CaCO_3_ particles used in thermoplastic compounds can be derived from natural mineral sources (calcite) or produced via chemical synthesis as precipitated calcium carbonate (PCC). However, the reinforcing efficiency of CaCO_3_ strongly depends on particle size, surface characteristics, and dispersion quality. Smaller particles with higher specific surface areas generally promote stronger interfacial interactions and more homogeneous dispersion within the polymer matrix [14,15].

Previous research has demonstrated the beneficial effect of CaCO_3_ on the dielectric behavior and aging resistance of PVC compounds [16,17,18,19]. In particular, our previous study [16] investigated the influence of combined UV–thermal aging on the electrical and dielectric properties of PVC/CaCO_3_ composites, with emphasis on dielectric response, electrical insulation performance, FTIR analysis, and physicochemical degradation mechanisms. Although the present work employs the same material system and accelerated aging protocol, its objectives and scope are fundamentally different. The focus of this study is on the evolution of the mechanical performance of PVC/CaCO_3_ composites, including yield stress, yield strain, stress at break, strain at break, tensile strength, and Young’s modulus, together with surface morphology (SEM–EDS and AFM), hydrophobicity, visual degradation, and carbonyl index analysis. These complementary experimental investigations were not reported in the previous publication. By correlating the mechanical response with microstructural, morphological, and surface modifications induced by prolonged UV–thermal exposure, the present study provides new experimental evidence and a more comprehensive understanding of the degradation mechanisms governing the long-term durability of PVC/CaCO_3_ composites. Accordingly, this work extends our previous research by offering a distinct and complementary perspective on the aging behavior of these materials.

## 2. Experimental Setup

The material investigated in this study was prepared at the ENICAB laboratory (Entreprise Nationale de l’Industrie du Câble, Biskra, Algeria). The base material consisted of commercial PVC 4000M resin, a standard formulation widely employed for wire and cable insulation. This resin is manufactured by the National Petrochemical Industries Company (ENIP, Skikda, Algeria) through the suspension polymerization process.

To evaluate the influence of inorganic filler content, calcium carbonate (CaCO_3_) was incorporated into the PVC matrix at concentrations of 2.5, 5.0, and 7.5 wt.%. The CaCO_3_ powder (Sigma-Aldrich, St. Louis, MO, USA; reference: 12010-1KG-R; Pcode: 102243766) was selected because of its high purity (approximately 99%) and fine particle size, with an average diameter below 50 μm. These characteristics promote a more homogeneous dispersion within the polymer matrix, which may affect the electrical properties and aging behavior of the composite through filler–matrix interfacial interactions. The relative permittivity of neat PVC is approximately 3.1, whereas that of calcium carbonate is about 6.1.

The PVC/CaCO_3_ composites were fabricated using a Polymix 200 P two-roll calendering mill (Brabender, Duisburg, Germany). The equipment features independently heated rollers, allowing precise control of the processing conditions. During processing, the roller temperature was maintained at approximately 170 °C to ensure adequate melting of the polymer. The PVC resin and the required amount of CaCO_3_ were premixed and simultaneously fed into the calender, where they were processed at a working temperature of about 165 °C for 15 min to obtain a uniform composite. The resulting sheets were then cut into specimens with dimensions of 50 mm × 50 mm and a thickness of approximately 1.4 mm for subsequent characterization.

**Figure 1:**
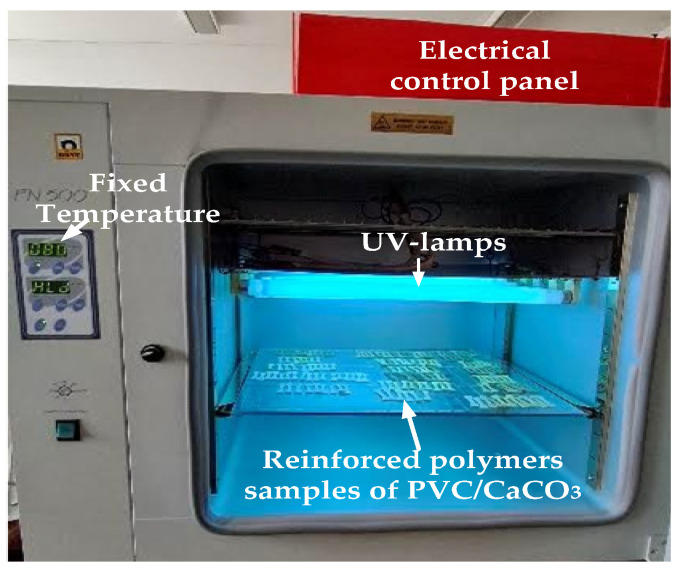
NUVE FN500 chamber used for simultaneous ultraviolet (UV) and thermal aging [16].

The accelerated aging experiment consisted of continuous UV–thermal exposure for a total duration of 1248 h (52 days) at 80 °C under dry, non-ventilated conditions. The specimens were continuously exposed to ultraviolet irradiation throughout the aging period, and no condensation, water spray, humidity, or dark recovery stages were applied. Therefore, the aging protocol was designed as a laboratory-controlled continuous UV–thermal exposure rather than a standardized cyclic weathering test according to ISO 4892-1 [20] or ASTM G154-23 [21]. The specimens were periodically removed every 96 h solely for inspection and characterization before being returned to the chamber. Thus, the 96 h intervals correspond to sampling intervals, not UV weathering cycles. This methodology is consistent with accelerated aging protocols used to evaluate the performance of PVC composites under harsh environmental conditions [16]. To evaluate the durability of the composites, accelerated aging was performed under combined UV radiation and elevated temperature in non-ventilated conditions using a NUVE experimental oven, model FN500 (Nuve, Ankara, Turkey). The apparatus is fitted with six 15 W Philips lamps, mounted in parallel and evenly spaced. The emitted radiation was predominantly in the UV-A range (≈350–400 nm), with a minor contribution from UV-B, consistent with established practices described for polymer photo-aging tests [15,16]. The PVC specimens filled with CaCO_3_ were placed at a fixed distance of 10 cm from the light sources. The chamber used for simultaneous ultraviolet (UV) and thermal aging is shown in Figure 1.

**Figure 2:**
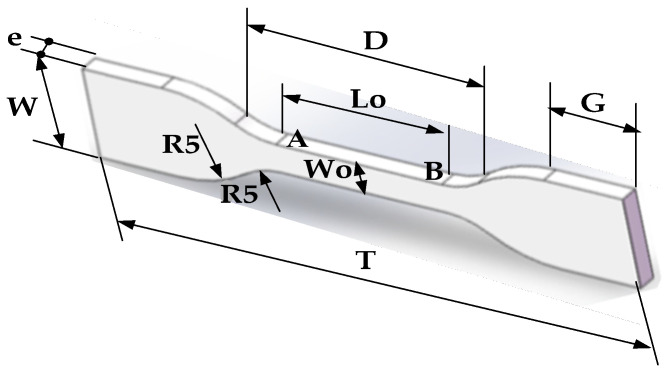
Tensile test piece of PVC and PVC/CaCO_3._

The specimens intended for mechanical testing (Figure 2) were molded in a dumbbell (dog-bone) shape (by the Electro-Industries Material Laboratory, Azazga, Algeria) with detailed dimensions given in Table 1, in accordance with standardized recommendations for tensile testing of plastics [22].

**Figure 3:**
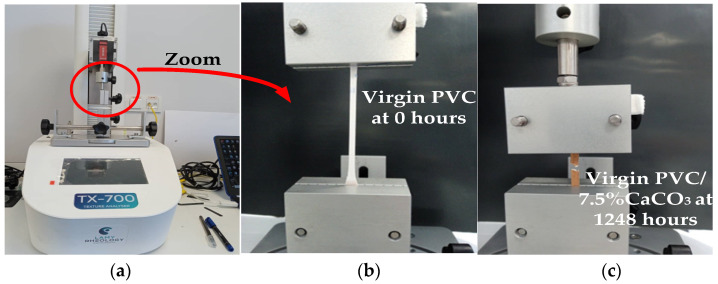
(**a**) Photo of the tensile test system (Texture Analyser, TX-700, Lamy Rheology, Champagne-au-Mont-d’Or, France), (**b**) Photo of the tensile test of the virgin PVC in elongation before break at 0 h, (**c**) Photo of the tensile test of the PVC/7.5CaCO_3_ at break after 1248 h UV.

Tensile tests (Figure 3) were performed under uniaxial tension in accordance with ISO 527-1:2019 [22] using a TEX-700 tensile testing system (Lamy Rheology, Champagne-au-Mont-d’Or, France) equipped with a touchscreen analyzer. The analyzer enables real-time display of stress–strain curves, data storage, and data transfer either directly or via an external computer through USB or RS232 connections. The system is compatible with Rheo Tex software (Lamy Rheology, Champagne-au-Mont-d’Or, France; TX-700 license Ref. N311000 + N311777) for test acquisition, while the experimental data were processed and analyzed using Origin 8 software (OriginLab Corporation, Northampton, MA, USA). Tests were conducted at a crosshead speed of 0.2 mm/s at room temperature (20 °C) using a 1 kN load cell. The specimens were dumbbell-shaped with a thickness e and total length T. For each composite formulation, the reported values represent the average of three specimens. The specimens were tested at room temperature (20 °C). The yield stress was determined as the maximum tensile stress obtained from the stress–strain curve, and Young’s modulus was calculated from the initial linear region of the curve. Contact angle, SEM–EDS and AFM characterization: Surface characterization of the PVC/CaCO_3_ composites was performed using contact angle measurements, scanning electron microscopy coupled with energy-dispersive X-ray spectroscopy (SEM–EDS), and atomic force microscopy (AFM). The surface wettability was evaluated using an optical goniometer operating according to the sessile drop method. A distilled water droplet of approximately 5 μL was deposited onto the specimen surface using a precision microsyringe. The droplet profile was recorded with a high-resolution digital camera and automatically analyzed using Autocad2022 (Autodesk, Inc., San Francisco, CA, USA) to determine the left and right contact angles and their average value. These measurements were used to assess the hydrophilic/hydrophobic behavior of the composites before and after UV–thermal aging and were carried out at Ferhat Abbas Sétif 1 University (UFAS1), Algeria. The surface morphology and elemental composition were SEM-EDX analyses were carried out using a Talos F200X scanning electron microscope (manufactured by Thermo Scientific, Waltham, MA, USA) equipped with an energy-dispersive X-ray spectroscopy (EDS) detector. Elemental analyses were performed using EDAX APEX software (Version 2.0), manufactured by EDAX LLC, Mahwah, New Jersey, USA at an accelerating voltage of 15 kV, a magnification of ×2718, an X-ray take-off angle of 34.9°, a live acquisition time of 100 s, and an amplification time of 7.68 μs. In addition, the surface topography of the composites was characterized using a Bruker Dimension Icon atomic force microscope (Bruker Corporation, Santa Barbara, CA, USA) equipped with a Dimension 4000 scanner (maximum scan area of 93 μm × 93 μm). AFM images were acquired over an area of 20 μm × 20 μm with a resolution of 256 × 256 pixels and processed using NanoScope Control Analysis software (Version 8.15) (Bruker Corporation, Santa Barbara, CA, USA). The carbonyl index values were calculated from the FTIR spectra reported in a previous study [16].

## 3. Results

### 3.1. Mechanical Properties Variation Throughout UV-Thermal Exposure

#### 3.1.1. Evolution of Stress–Strain Parameters Under UV–Thermal Aging

The mechanical properties of virgin PVC and PVC/CaCO_3_ composites subjected to UV–thermal aging at different exposure times are summarized in Table 2. UV–thermal aging progressively modified the mechanical behavior of all formulations. The most pronounced effect was observed for the strain at break (ε_max_), which decreased continuously with increasing exposure time, indicating a progressive loss of ductility and flexibility. Similarly, the yield strain (ε_y_) decreased during aging, confirming the increasing brittleness of the material.

The yield stress (σ_y_) exhibited only moderate variations throughout the aging period. A slight increase was observed during the early stages of exposure (up to approximately 96 h) for some formulations, suggesting that limited crosslinking and molecular rearrangements temporarily reinforced the polymer network. However, with prolonged UV–thermal exposure, chain scission and oxidative degradation became the dominant mechanisms, resulting in a gradual reduction in yield stress and mechanical performance [15,16,23].

The stress at break (σ_max_) remained relatively stable throughout the aging period, exhibiting only moderate fluctuations depending on the CaCO_3_ content and exposure time. In contrast, the continuous reduction in strain at break demonstrates that UV–thermal aging primarily affected the deformation capability of the composites rather than their ultimate load-bearing capacity.

The incorporation of CaCO_3_ influenced the degradation behavior of PVC during UV–thermal aging. Compared with unfilled PVC, the filled composites generally exhibited better retention of strain at break and more stable stress values during prolonged exposure, indicating that the filler partially mitigated the effects of photo-oxidative degradation. This behavior can be attributed to the restriction of polymer-chain mobility and the barrier effect provided by well-dispersed CaCO_3_ particles. However, increasing the filler loading to 7.5 wt% did not produce a proportional improvement, probably because particle agglomeration and local stress concentrations reduced the effectiveness of the reinforcement.

#### 3.1.2. Tensile Strength, Elongation at Break and Young’s Modulus Analysis According to UV-Thermal Exposure Time

**Figure 4:**
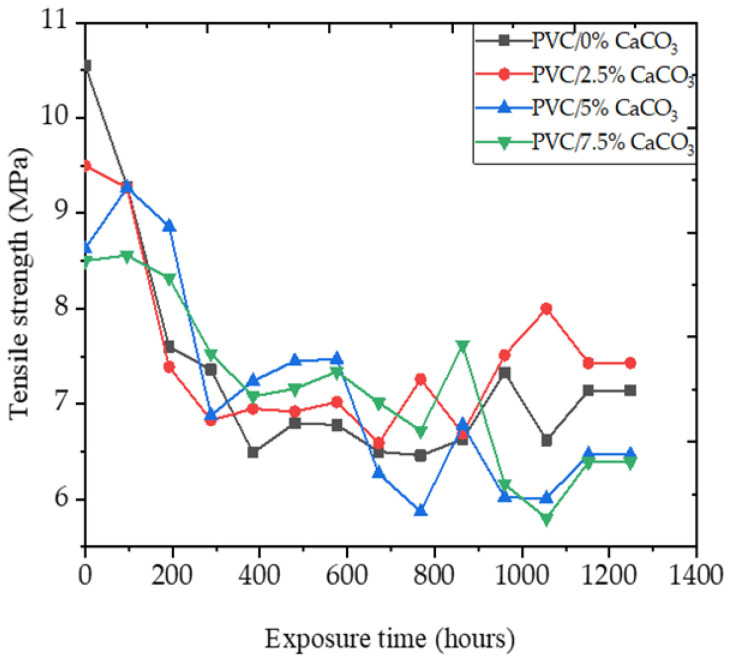
Evolution of tensile strength of PVC and PVC/CaCO_3_ composites as a function of UV–thermal exposure time.

**Figure 5:**
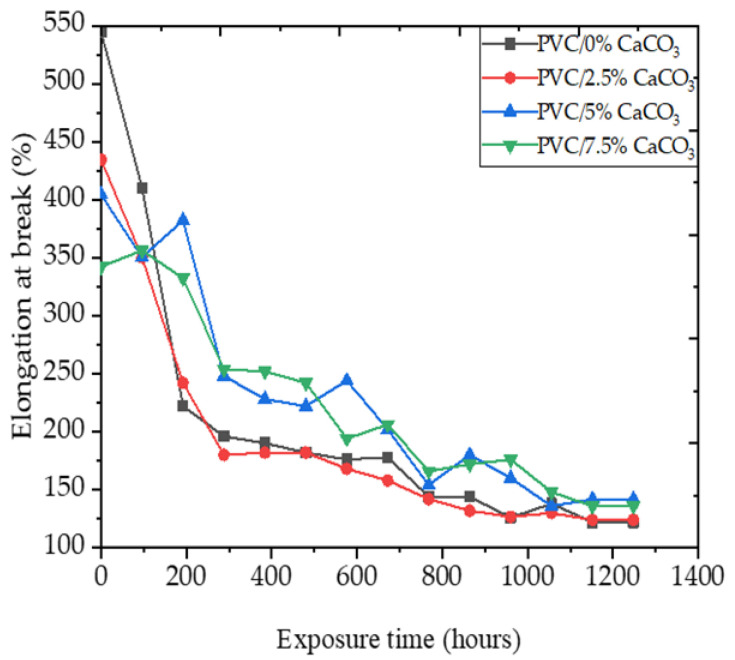
Evolution of elongation at break of PVC and PVC/CaCO_3_ composites as a function of UV–thermal exposure time.

**Figure 6:**
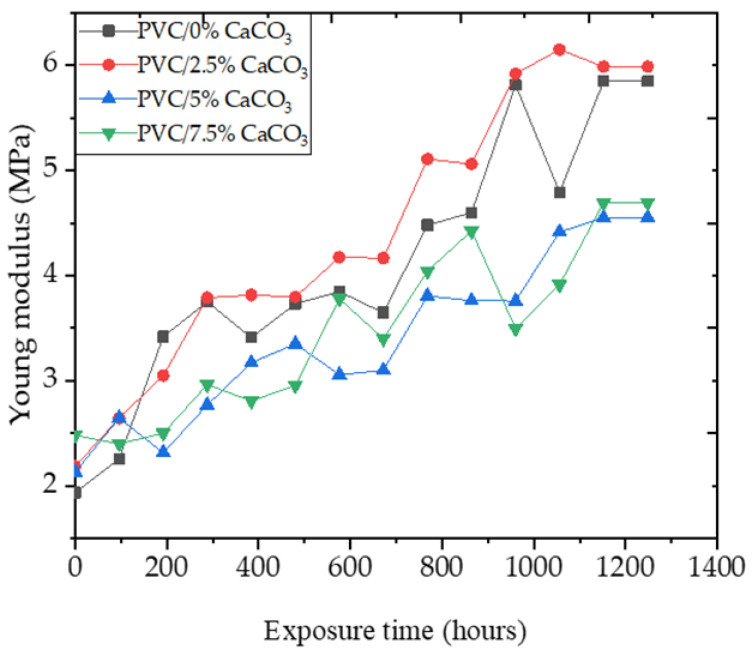
Evolution of Young’s modulus of PVC and PVC/CaCO_3_ composites as a function of UV–thermal exposure time.

Figure 4, Figure 5 and Figure 6 were obtained using the complete experimental dataset obtained at 96-h inspection intervals throughout the 1248-h UV–thermal aging experiment.

As shown in Figure 4, the evolution of tensile strength with UV–thermal exposure indicates a rapid reduction during the early stages of aging, followed by a more gradual variation at longer exposure times. Overall, the incorporation of CaCO_3_ generally improves the retention of tensile strength during UV–thermal aging compared with unfilled PVC, although the extent of this improvement depends on both filler loading and exposure time. Among the investigated formulations, the 2.5 wt% CaCO_3_ composite exhibits the best retention of tensile strength during prolonged aging, indicating that this filler content is particularly effective in limiting the loss of mechanical strength. The 5 wt% formulation also maintains relatively stable tensile strength throughout the exposure period, while the 7.5 wt% formulation displays greater fluctuations during prolonged aging. This behavior is consistent with the possible formation of filler agglomerates, which may promote localized stress concentrations and increase the variability of the mechanical response [24]. These observations suggest that moderate CaCO_3_ loadings (2.5–5 wt%) are more effective than the highest filler loading in preserving tensile strength during long-term UV–thermal aging. The observed behavior is presented as a qualitative description of the experimental data rather than as the result of a formal kinetic analysis.

As shown in Figure 5, the evolution of elongation at break with UV–thermal exposure exhibits a pronounced reduction during the early stages of aging, followed by a more gradual variation at longer exposure times. The progressive decrease in elongation at break indicates a gradual loss of ductility associated with UV–thermal degradation of the PVC matrix [25]. Although all formulations exhibit this general trend, the CaCO_3_-filled composites show improved retention of elongation at break compared with unfilled PVC during the later stages of aging, particularly for the formulations containing 5 and 7.5 wt% CaCO_3_. In contrast, the unfilled PVC exhibits the most pronounced reduction in ductility throughout the exposure period.

As shown in Figure 6, the evolution of Young’s modulus with UV–thermal exposure exhibits a general and progressive increase throughout the aging period. This interpretation is based on the experimental trends obtained from measurements performed at 96-h inspection intervals during the 1248-h aging experiment. The increase in Young’s modulus indicates the gradual stiffening of the PVC composites during UV–thermal aging, which may be attributed to physical aging and limited photo-induced crosslinking occurring during the early stages of exposure [26]. During the initial stages of aging, the differences among the various formulations remain relatively small. However, as the exposure time increases, the distinction between the lower filler contents (0 and 2.5 wt%) and the higher filler contents (5 and 7.5 wt%) becomes more pronounced, with the lower filler-content composites exhibiting higher Young’s modulus values at the end of the aging period.

### 3.2. Hydrophobicity Analysis: Static Contact Angle

**Figure 7:**
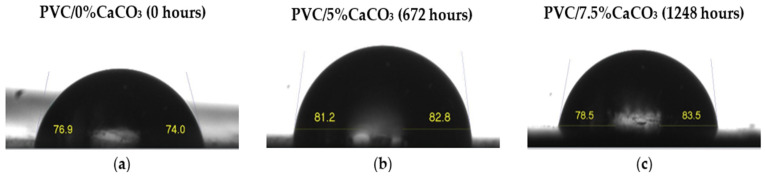
Contact angles in hydrophobicity test: (**a**) Virgin PVC 0 h, (**b**) PVC/5%CaCO_3_ at 672 h, and (**c**) PVC/7.5%CaCO_3_ at 1248 h.

The influence of CaCO_3_ fillers on the hydrophobicity of PVC composites was evaluated by measuring the static contact angle of a water droplet of 0.5 μL on the specimen surface. The hydrophobicity investigation was primarily intended to provide a qualitative assessment of the evolution of the surface wettability of the PVC/CaCO_3_ composites during UV–thermal aging. Consequently, Figure 7 presents representative images selected to illustrate the progressive changes in surface behavior at characteristic aging stages rather than the complete set of measurements. Specifically, virgin PVC at 0 h was chosen as the reference material, the PVC/5 wt% CaCO_3_ specimen after 672 h represents an intermediate stage of aging, and the PVC/7.5 wt% CaCO_3_ specimen after 1248 h illustrates the material after prolonged exposure. These representative examples were selected because they clearly demonstrate the evolution of the surface condition and the beneficial influence of CaCO_3_ on maintaining hydrophobicity during aging.

- The unfilled specimen exhibits lower initial hydrophobicity (~77°).
- The addition of 5 wt% CaCO_3_ increases the contact angle (~83°), indicating improved hydrophobicity, even after exposure time of 672 h.
- At 7.5 wt% CaCO_3_ and after 1248 h exposure time, the contact angle remains high (~84°), showing good stability and significant improvements.

Finally, the incorporation of CaCO_3_ enhances hydrophobicity, with an optimal effect observed between 5 wt% and 7.5 wt%.

Prolonged UV–thermal exposure modifies both the surface morphology and the surface chemistry of the PVC composites. Although AFM observations (Section 3.4) indicate an increase in surface roughness after aging, the evolution of the water contact angle cannot be attributed to roughness alone. According to the Wenzel wetting model, surface roughness amplifies the intrinsic wettability of a material, increasing the contact angle on hydrophobic surfaces and decreasing it on hydrophilic surfaces [27]. Simultaneously, oxidative aging generates oxygen-containing polar functional groups and increases surface heterogeneity, while possible additive migration may further alter the surface free energy [28]. Consequently, the measured contact angle reflects the combined influence of surface roughness and chemical modifications [29]. The incorporation of CaCO_3_ helps retard these changes, thereby improving the retention of surface hydrophobicity [30].

### 3.3. SEM-EDS Analysis

**Figure 8:**
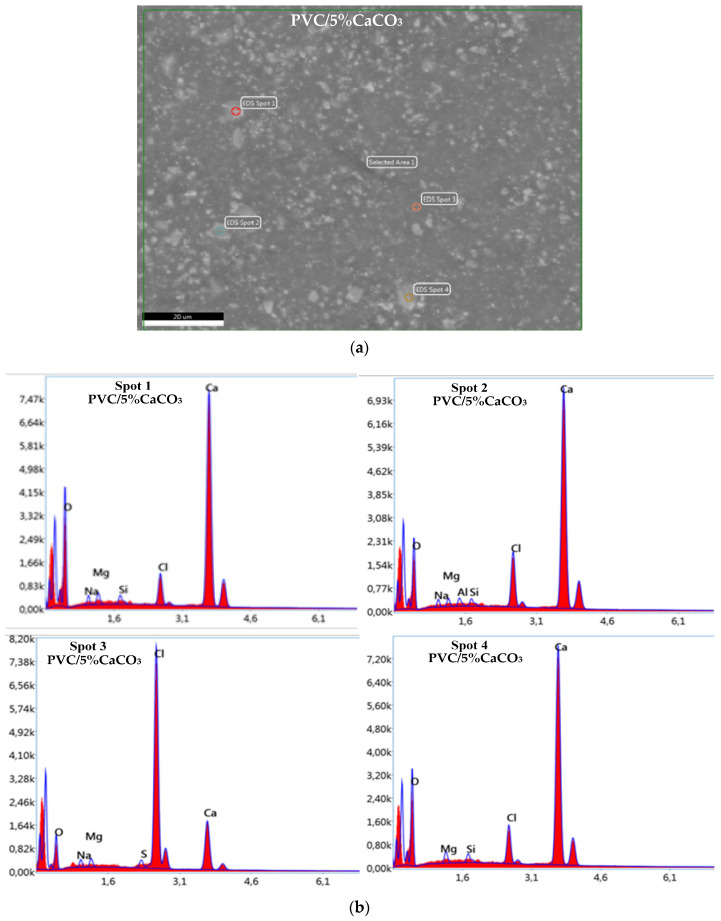
(**a**) Locations of SEM–EDS analysis points (spots 1–4), (**b**) SEM-EDS of spots.

Figure 8a shows the locations of the analyzed spots in the micrographs, while the corresponding SEM–EDS spectra are presented in Figure 8b. The results indicate that the composite microstructure remains more homogeneous and less damaged after prolonged exposure, particularly at higher filler contents. Among the tested materials, the PVC/5 wt% CaCO_3_ composite at the initial stage of aging exhibits the best overall performance, combining improved chemical stability and morphological integrity in agreement with previous studies [16,31].

The SEM–EDS spectra (Figure 8b) confirm the presence of a CaCO_3_-rich phase within the composite. Besides the major elements associated with the PVC matrix and the CaCO_3_ filler, small amounts of Na, Mg, Si, Al, S, and Cl were also detected in localized regions [32,33]. Chlorine is an intrinsic constituent of the PVC matrix, whereas the minor sulfur signal may be associated with additives in the commercial PVC formulation, trace impurities in the raw materials, or local compositional heterogeneities. Since SEM–EDS provides only elemental identification, the exact origin of these minor elements cannot be determined from the present analysis alone. Overall, the material exhibits a CaCO_3_-rich matrix, but with local heterogeneities marked by inclusions or contaminants [34].

### 3.4. Morphological Examination and Hydrophobicity of UV-Thermal Irradiated Specimens

Atomic Force Microscopy (AFM) is an emerging non-destructive technique for characterizing surface topography and investigating morphological and structural properties at the micro- and submicrometric scales. Scanning the surface with the AFM tip provides a spatial profile or map of the specimen region, which is used to gather surface/tip interaction data, roughness and pore size distributions, which play an important role in the tensile strength of PVC/CaCO_3_ composites. The specimens analyzed were square-shaped regions with lateral dimensions of 20 μm.

**Figure 9:**
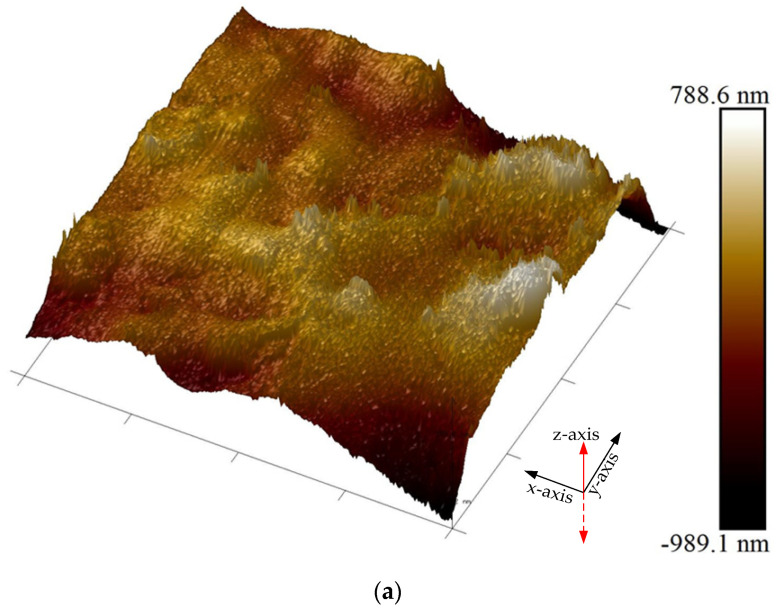

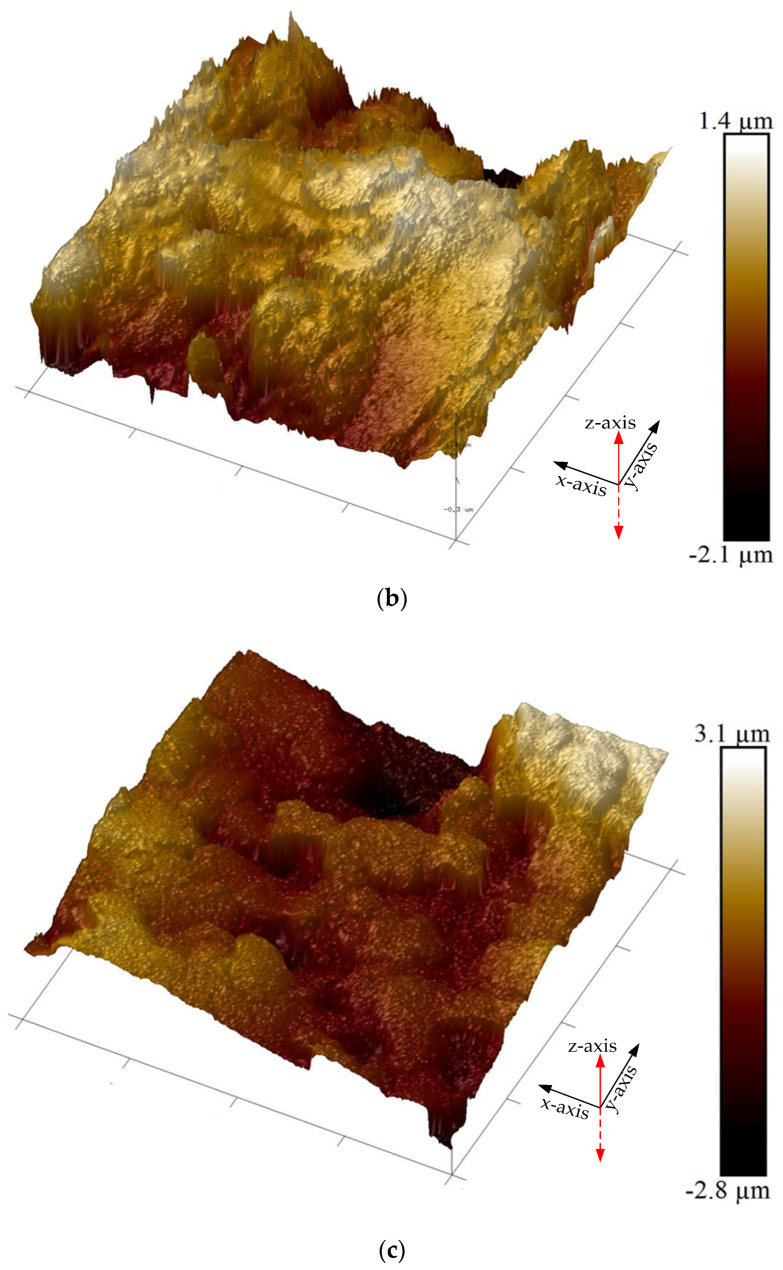
AFM surface morphology of PVC/CaCO_3_ composites: (**a**) virgin PVC (0 h), (**b**) PVC/5 wt% CaCO_3_ after 672 h, and (**c**) PVC/7.5 wt% CaCO_3_ after 1248 h of UV–thermal aging.

AFM analyses were performed on the surfaces of virgin PVC and PVC/CaCO_3_ polymers before and after aging to evaluate surface modifications induced by photo-oxidative degradation. Representative micrographs of PVC/CaCO_3_ films in the virgin state and after 672 and 1248 h of aging are depicted in Figure 9.

For virgin PVC, the surface (Figure 9a) appears smooth with minimal imperfections and no significant textural features. After 672 h of UV-Thermal exposure, the surface of the PVC/5%CaCO_3_ composite (Figure 9b) exhibited the formation of linear cracks (highlighted brown), suggesting the onset of degradation and supporting earlier observations.

After 1248 h of aging, the surface of the PVC/7.5%CaCO_3_ composite (Figure 9c) showed increased roughness and more pronounced features, such as circular and irregular depressions, which suggested advanced surface degradation. This structural alteration indicates that prolonged exposure weakens the material, leading to cavity formation in the most degraded regions.

Larger fractures and fissures are observed, indicating severe structural failure. The photographs demonstrate widespread crack propagation and extreme surface roughness. This rough, broken surface form is typical of UV-exposed materials, which are brittle and unstable. As aging progresses, PVC/CaCO_3_ composites—particularly those with lower filler contents—develop surface pits and increased roughness, leading to enhanced brittleness under UV–thermal aging conditions. This differential in deterioration morphology may influence practical applications, as composites with higher CaCO_3_ loading may remain more flexible under UV-thermal exposure. It should be noted that the AFM analysis was performed as a qualitative assessment of the surface morphology. Accordingly, the micrographs presented in Figure 9 correspond to representative specimens selected to illustrate characteristic stages of the degradation process rather than to provide a systematic comparison of all CaCO_3_ contents and aging durations. Therefore, the observed differences in surface roughness, crack formation, and cavity development should be interpreted as the combined effects of UV–thermal aging and filler incorporation [35]. The AFM observations alone do not allow the individual contributions of aging time and CaCO_3_ content to be separated. Instead, they complement the mechanical, hydrophobicity, and SEM–EDS analyses, which together provide a more comprehensive understanding of the degradation behavior of the PVC/CaCO_3_ composites.

### 3.5. Visual Observation of Photo-Discoloration Under UV-Thermal Exposure

**Figure 10:**
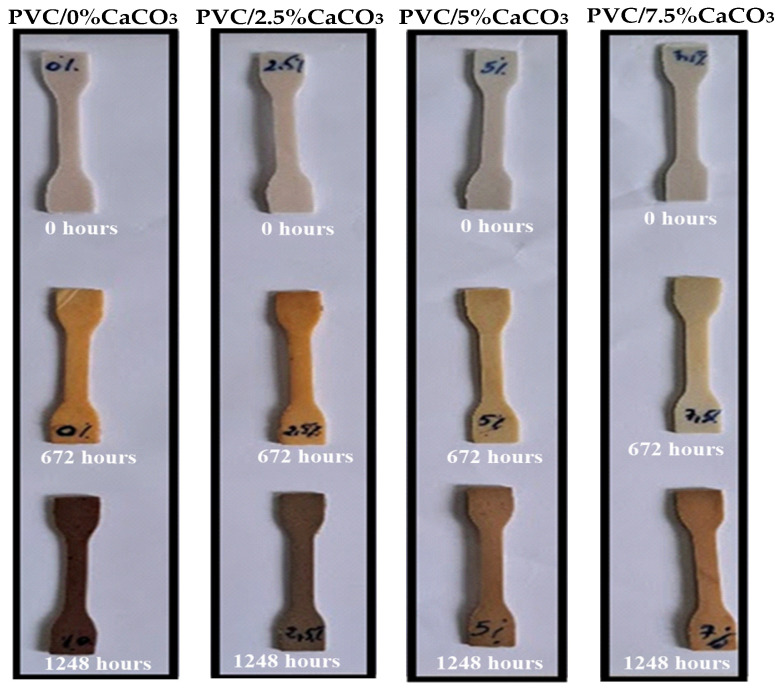
Changes in the texture and coloration under UV-thermal exposure times (0 h, 672 h and 1248 h).

Figure 10 illustrates the changes in texture and color of dumbbell-shaped PVC specimens containing 0, 2.5, 5, and 7.5 wt% CaCO_3_, subjected to combined UV and thermal stresses.

The visual examination of PVC specimens containing 0, 2.5, 5, and 7.5 wt% CaCO_3_ reveals a progressive change in appearance with aging time (0, 672, and 1248 h). At the beginning of exposure (0 h), all specimens exhibit a homogeneous, smooth surface with no visible defects, reflecting the non-degraded initial state. After 672 h of exposure, slight changes become noticeable: progressive yellowing and darkening of the material surface, a loss of transparency of unfilled PVC, and microcracking or matte zones on filled specimens, with intensity increasing as CaCO_3_ loading decreased. The observed discoloration could reflect oxidation phenomena induced by combined aging, as prolonged exposure may lead to an increased absorption of high-energy photons by the PVC, thereby enhancing chain scission mechanisms and promoting the formation of chromophore groups [16,36]. The PVC specimens with 5 and 7.5 wt% CaCO_3_ content maintain good surface uniformity, whereas unfilled PVC shows more pronounced discoloration. After 1248 h of exposure, the differences become more evident. Unfilled PVC shows severe yellowing to browning, accompanied by a loss of surface uniformity. In contrast, specimens with 5 and 7.5 wt% CaCO_3_ display improved visual stability, with more moderate discoloration and relatively compact surfaces. Specimens with lower filler contents (0 and 2.5 wt%) exhibit more advanced degradation, characterized by whitish regions, cracking, and increased surface heterogeneity, indicating faster embrittlement and less effective stabilization.

### 3.6. Carbonyl Index Calculation

The carbonyl index (CI) was used to evaluate the extent of photo-oxidative degradation of the PVC/CaCO_3_ composites during UV–thermal aging. The CI was calculated from the Fourier transform infrared (FTIR) spectra as the ratio between the absorbance of the carbonyl band at 1720 cm^−1^ and that of the reference C–Cl band at 610 cm^−1^, according to:

(1) $$CI=\frac{I_{1720}}{I_{610}}$$

where I_1720_ corresponds to the absorbance intensity of the carbonyl (C=O) stretching vibration generated during oxidation, and I_610_ is the absorbance intensity of the C–Cl stretching vibration, which is used as an internal reference because it remains comparatively stable during the degradation process.

**Figure 11:**
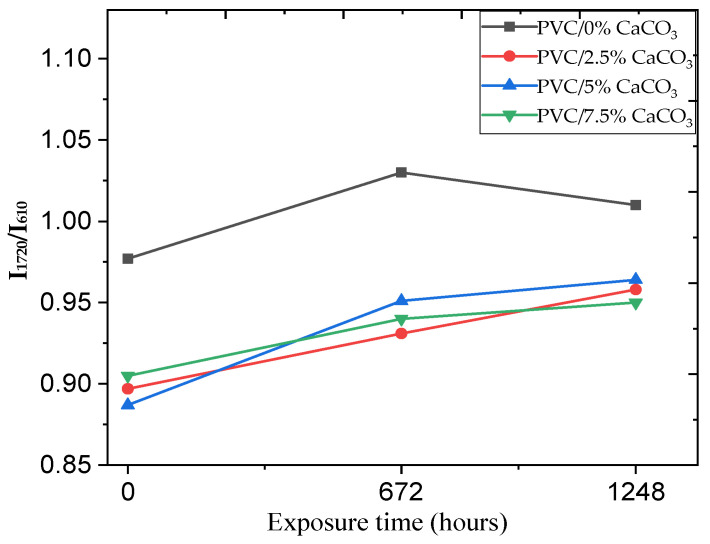
Carbonyl index of PVC and PVC/f under UV-thermal exposure times (0 h, 672 h, 1248 h).

Figure 11 shows the evolution of the measured parameter as a function of UV–thermal aging time for PVC composites containing different CaCO_3_ loadings. The unfilled PVC (0 wt% CaCO_3_) exhibits the highest values throughout the aging period, with an initial increase up to the intermediate exposure time, followed by a slight decrease after prolonged aging. In contrast, all CaCO_3_-filled composites display lower initial values but a gradual increase with increasing exposure time. Among the filled formulations, the 5 wt% CaCO_3_ composite exhibits the greatest increase during aging and reaches the highest final value, while the 2.5 wt% and 7.5 wt% CaCO_3_ composites show similar trends with slightly lower values. These results indicate that UV–thermal aging affects the different formulations differently and suggest that the incorporation of 5 wt% CaCO_3_ provides the most favorable response among the filled composites for the measured property. The increase in the carbonyl index reflects the formation of oxygen-containing oxidation products and therefore indicates the progression of oxidative degradation [37,38,39].

### 3.7. Degradation Mechanism Scheme

**Figure 12:**
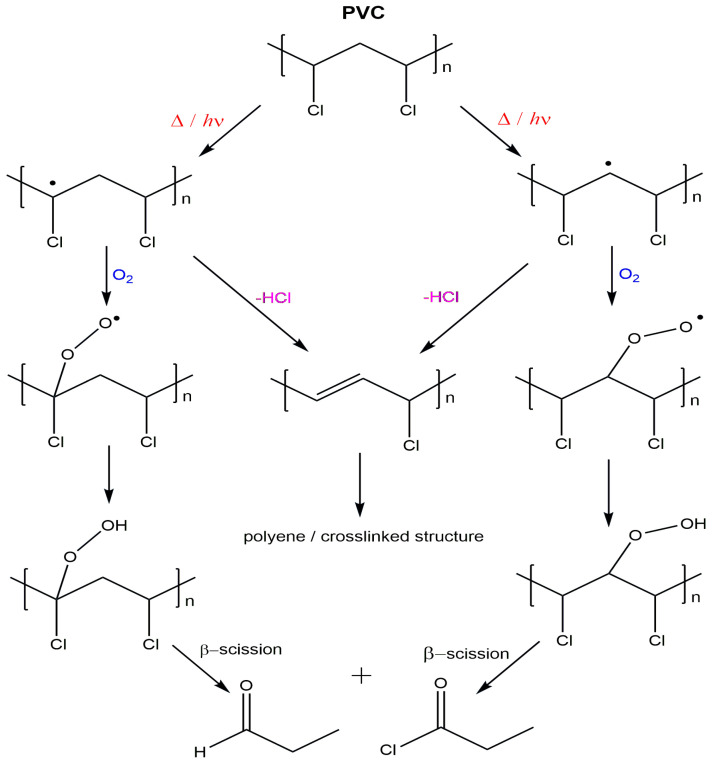
Schematic illustration of the simplified principal structural decomposition pathways of PVC under thermal and/or UV aging.

During the structural degradation of PVC, various reactions may occur depending on the environmental conditions. PVC degradation is a complex phenomenon; thus, tracking these reactions is difficult because they go through free-radical mechanisms. However, structural degradation of PVC should basically be evaluated in two different situations. One is chain scissions and/or crosslinking that occur rapidly at high temperatures, and the other is slowly progressing degradation under weathering at low temperatures. Gardi et al. recently reviewed the weathering of PVC, degradation mechanisms and durability of PVC parts [40]. They indicated that mainly two kinds of reactions take place in PVC parts when they are exposed to UV light. The first one is thermal dehydrochlorination, generating conjugated polyene sequences at high temperatures by releasing HCl and yielding unsaturated C=C bonds in the backbone. It is known that the dehydrochlorination of PVC is an autocatalytic reaction and occurs by the unzipping mechanism. Yellowing, then a rapid darkening in color, is another simple indicative change of thermal dehydrochlorination of PVC. Double bond formation could also initiate crosslinking reactions, and the aged part quickly becomes brittle. A second series of reactions involves oxygen, and it causes severe photo-oxidation, bringing about chain scission and the formation of carbonyls. Figure 12 schematizes a simplified version of these two main structural decomposition reactions of PVC, induced by heat and/or UV aging and proceeds toward polyene and/or crosslinking formation and b-scission. In this study, it has been shown and concluded that CaCO_3_ microparticles could successfully mitigate PVC degradation with the following effects: (i) neutralization of the released HCl, (ii) inhibition of radical propagation, (iii) reduction of oxygen diffusion into the polymer matrix, (iv) partial reflection and scattering of UV radiation, and (v) improved heat dissipation, thereby reducing localized hot spots.

## 4. Discussion

The combined mechanical, morphological, and surface analyses indicate that UV–thermal exposure progressively degrades PVC through photo-oxidative and thermo-oxidative mechanisms, leading to chain scission, embrittlement, discoloration, and surface cracking. The addition of CaCO_3_ mitigates these degradation processes by restricting polymer chain mobility, reducing oxygen diffusion, and partially shielding the polymer from UV radiation. However, the beneficial effect depends on filler content. Moderate loadings (around 5 wt%) provide the best balance between mechanical reinforcement and long-term stability, whereas higher loadings may promote particle agglomeration and localized stress concentrations, reducing the effectiveness of the reinforcement. The agreement between the tensile, AFM, SEM–EDS, hydrophobicity, and carbonyl index results confirm that mechanical degradation is closely linked to the evolution of the material’s microstructure and surface chemistry during aging.

## 5. Conclusions

This study investigated the effect of CaCO_3_ loading on the mechanical performance, surface characteristics, and long-term durability of PVC composites subjected to prolonged combined UV and thermal aging. By combining mechanical testing with AFM, SEM–EDS, contact angle measurements, and carbonyl index analysis, a comprehensive assessment of the degradation behavior was achieved. The results provide a better understanding of the mechanisms governing UV–thermal aging and the role of CaCO_3_ in improving the durability of PVC composites. The main conclusions are as follows:

- UV–thermal aging progressively degraded the PVC matrix, resulting in increased stiffness, reduced ductility, surface cracking, discoloration, and overall deterioration of the mechanical properties.
- The incorporation of CaCO_3_ improved the resistance of PVC to UV–thermal degradation by enhancing mechanical stability, preserving surface integrity and hydrophobicity, and slowing the progression of photo-oxidative damage.
- Among the investigated formulations, approximately 5 wt% CaCO_3_ provided the best overall balance between mechanical performance, durability, and long-term aging resistance, whereas higher filler loading did not provide additional benefits.
- The combined mechanical, AFM, SEM–EDS, contact angle, and carbonyl index analyses consistently demonstrated that CaCO_3_ delays degradation by improving the microstructural and surface stability of the composites during prolonged exposure.
- Overall, the results demonstrate that optimizing the CaCO_3_ content is essential for maximizing the long-term performance of PVC composites and support the use of PVC/CaCO_3_ materials in outdoor applications requiring enhanced environmental durability and service life.

## Figures and Tables

**Table 1:** Dimensions of the tensile test pieces.

Dimension Designation	Value (mm)
Width of the narrow section (W_o_)	4.25
Width overall (W)	9
Length of narrow section (D)	20
Length overall (T)	50
Gauge length (L_o_)	15
Border overall length (G)	10
Thickness (e) (average)	1.5

**Table 2:** Maximum values of stress and strain according to UV-thermal exposure.

Exposure Time (h)		0	96	192	288	384	480	576	672	768	864	960	1056	1248
Characteristic	CaCO_3_ (wt%)													
Yield stress (MPa): **σ_y_**	**0**	7.0	7.5	7.25	6.8	6.7	6.5	6	4	4.5	4	3.5	3.25	3.15
	**2.5**	6.8	7.8	7.5	6	7	6.5	5.8	4	4.8	6	5.8	4	4.5
	**5**	6	7.8	7	6	5.8	6	5	4	4.2	5	4.5	4	3.3
	**7.5**	6.5	6.2	6.5	7	6.5	6	5.8	6	5	4.8	4.5	4	3.5
Yield strain (%): **ε_y_**	**0**	200	200	190	175	160	140	142	125	110	108	105	103	102
	**2.5**	210	205	180	170	180	160	140	120	115	140	130	115	105
	**5**	180	200	180	170	168	145	120	140	118	120	115	110	105
	**7.5**	180	170	180	190	185	160	150	150	135	120	118	112	105
Stress at break (MPa): **σ_max_**	**0**	10.3	9.3	7.6	7.4	6.5	7.1	6.8	6.6	7.0	7.5	7.0	6.9	7.2
	**2.5**	9.5	9.4	8	6.9	7.6	7.5	6.2	7.2	6.6	7.2	6.6	6.1	7.1
	**5**	8.6	9.4	8.9	6.9	7.6	7.5	6.2	7.2	6.6	7.2	6.6	6.1	7.1
	**7.5**	8.5	8.6	8.6	7.8	7.3	7.5	7.4	7.2	6.8	6.9	6.6	6.4	6.5
Strain at break (%): **ϵ_max_**	**0**	545	410	222	196	190	178	176	140	138	124	134	134	120
	**2.5**	433	344	228	242	220	190	148	170	154	170	152	134	140
	**5**	401	344	378	242	220	190	152	170	152	170	152	134	140
	**7.5**	342	355	330	284	242	236	192	206	166	164	170	146	136

## Data Availability

The original contributions presented in this study are included in the article. Further inquiries can be directed to the corresponding author.
